# Impact of Neoadjuvant Bevacizumab on Neuroradiographic Response and Histological Findings Related to Tumor Stemness and the Hypoxic Tumor Microenvironment in Glioblastoma: Paired Comparison Between Newly Diagnosed and Recurrent Glioblastomas

**DOI:** 10.3389/fonc.2022.898614

**Published:** 2022-06-17

**Authors:** Jun Takei, Nei Fukasawa, Toshihide Tanaka, Yohei Yamamoto, Ryota Tamura, Hikaru Sasaki, Yasuharu Akasaki, Yuko Kamata, Mutsunori Murahashi, Masayuki Shimoda, Yuichi Murayama

**Affiliations:** ^1^ Department of Neurosurgery, Jikei University School of Medicine, Tokyo, Japan; ^2^ Department of Pathology, Jikei University School of Medicine, Tokyo, Japan; ^3^ Department of Neurosurgery, Jikei University School of Medicine Kashiwa Hospital, Kashiwa, Japan; ^4^ Department of Neurosurgery, Jikei University School of Medicine Daisan Hospital, Tokyo, Japan; ^5^ Department of Neurosurgery, Keio University School of Medicine, Tokyo, Japan; ^6^ Division of Oncology, Research Center for Medical Sciences, Jikei University School of Medicine, Tokyo, Japan

**Keywords:** bevacizumab, glioblastoma, FOXM1, magnetic resonance imaging (MRI), tumor microenvironment, pseudo-papillary structure

## Abstract

**Background:**

Previously, we reported that bevacizumab (Bev) produces histological and neuroradiographic alterations including changes in tumor oxygenation, induction of an immunosupportive tumor microenvironment, and inhibition of stemness. To confirm how those effects vary during Bev therapy, paired samples from the same patients with newly diagnosed glioblastoma (GBM) who received preoperative neoadjuvant Bev (neoBev) were investigated with immunohistochemistry before and after recurrence.

**Methods:**

Eighteen samples from nine patients with newly diagnosed GBM who received preoperative neoBev followed by surgery and chemoradiotherapy and then autopsy or salvage surgery after recurrence were investigated. The expression of carbonic anhydrase 9 (CA9), hypoxia-inducible factor-1 alpha (HIF-1α), nestin, and Forkhead box M1 (FOXM1) was evaluated with immunohistochemistry.

For comparison between neoBev and recurrent tumors, we divided the present cohort into two groups based on neuroradiographic response: good and poor responders (GR and PR, respectively) to Bev were defined by the tumor regression rate on T1-weighted images with gadolinium enhancement (T1Gd) and fluid-attenuated inversion recovery images. Patterns of recurrence after Bev therapy were classified as cT1 flare-up and T2-diffuse/T2-circumscribed. Furthermore, we explored the possibility of utilizing FOXM1 as a biomarker of survival in this cohort.

**Results:**

A characteristic “pseudo-papillary”-like structure containing round-shaped tumor cells clustered adjacent to blood vessels surrounded by spindle-shaped tumor cells was seen only in recurrent tumors. Tumor cells at the outer part of the “pseudo-papillary” structure were CA9-positive (CA9+)/HIF-1α+, whereas cells at the inner part of this structure were CA9−/HIF-1α+ and nestin+/FOXM1+. CA9 and HIF-1α expression was lower in T1Gd-GR and decreased in the “T2-circumscribed/T2-diffuse” pattern compared with the “T1 flare-up” pattern, suggesting that tumor oxygenation was frequently observed in T1Gd-GR in initial tumors and in the “T2-circumscribed/T2-diffuse” pattern in recurrent tumors. FOXM1 low-expression tumors tended to have a better prognosis than that of FOXM1 high-expression tumors.

**Conclusion:**

A “pseudo-papillary” structure was seen in recurrent GBM after anti-vascular endothelial growth factor therapy. Bev may contribute to tumor oxygenation, leading to inhibition of stemness and correlation with a neuroimaging response during Bev therapy. FOXM1 may play a role as a biomarker of survival during Bev therapy.

## Introduction

Bevacizumab (Bev) is a monoclonal antibody against vascular endothelial growth factor (VEGF)/vascular permeability factor and blocks endothelial proliferation and vascular permeability, thus reducing enhancement and perifocal edema in glioblastoma (GBM). The effect of Bev on GBM depends on not only inhibition of tumor angiogenesis but also alteration of the tumor microenvironment (TME) from immunosuppressive to immunosupportive (1).

The alteration in the TME induces tumor oxygenation from a hypoxic TME, leading to inhibition of stemness in the perivascular niche (2–4) and infiltration of immunosuppressive cells including regulatory T cells and M2 tumor-associated macrophages (5, 6). Although these effects including an immunosupportive TME are sustained for a long time, improvement in tumor oxygenation is transient (3), indicating that the therapeutic efficacy of Bev is difficult to maintain over a long period of time. Bev induces oxygenation of the TME, leading to tumor dormancy. A hypoxic TME restimulates stemness, which may be a reason for the dismal clinical outcome of GBM in a short period of time (7). The duration of maintenance of the TME in a dormant state may impact the clinical outcome, regardless of the initial response to Bev on gadolinium-enhanced neuroimages and perifocal edema.

In recent years, glioma stem cells (GSCs) have become a cell type of increasing interest. GSCs survive in hypoxic and starvation conditions (8, 9). A number of molecular markers are generally used to isolate and characterize GSCs (10, 11). The TME, including vascularity and tumor oxygenation, is very important for the survival of GSCs. More importantly, GSCs are resistant to radiation (RT) and temozolomide (TMZ) compared with differentiated tumor cells (12). In addition, a hypoxic TME induces VEGF expression, resulting in resistance to RT and TMZ and difficulty in controlling GSCs in recurrent tumors (13, 14).

CD133-positive (CD133+) cells include vascular endothelial cells and other cells in the perivascular niche that maintain GSC characteristics *via* VEGF and NOTCH signaling in the microenvironment (2). Bev is considered to be a reasonable treatment to control GSCs and maintain an oxygenated and immunosupportive TME. However, its efficacy is transient, and the biomarkers that predict survival remain unknown.

Among these molecular markers, nestin is an intermediate filament protein expressed in neural progenitor stem cells (15). Nestin is expressed in many GBMs, and the differentiation of GBM cells leads to the downregulation of nestin, a potential marker for GSCs (16). Whether the level of nestin expression is correlated with the histological grade of malignancy in gliomas and the clinical outcome is still controversial (17–19). Therefore, whether nestin expression is a biomarker for survival is uncertain.

We also focused on the possibility that Forkhead box M1 (FOXM1) may be a biomarker of survival during Bev therapy. FOXM1 is a key transcription factor, plays a critical role in tumorigenesis and transformation of normal astrocytes, and is overexpressed in GBM (20). FOXM1 also binds to the *VEGF* promoter and contributes to angiogenesis and growth of GSCs in GBM by upregulation of VEGF (21). Furthermore, FOXM1 is upregulated in recurrent GBM, both at the mRNA and protein levels, and a high level of FOXM1 expression is associated with poor prognosis in recurrent GBM (22). However, no previous studies have investigated alterations in FOXM1 expression or its reliability as a predictive biomarker of survival in GBM during anti-VEGF therapy.

Previously, we reported that Bev induces tumor oxygenation in accordance with a decrease in microvessel density (MVD) and inhibition of immunosuppressive cell and stem cell infiltration by comparative analyses among initial GBM (naive-Bev), GBM with radiological effectiveness of Bev at the time of treatment with preoperative neoadjuvant Bev (neoBev, effective-Bev), and recurrent GBM after Bev failure (refractory-Bev) (3, 5). However, in our previous studies, all samples of refractory-Bev were derived from patients who did not receive neoBev.

As far as we know, the present study is the first report to investigate the neuroradiographic response before and after recurrence with a comparison of paired samples from the same patients who received preoperative neoBev followed by surgery combined with RT, TMZ, and Bev, and then autopsy or salvage surgery after Bev failure. The purpose of this study was to investigate the following: 1) the difference between neoBev and refractory-Bev according to histological and immunohistochemical findings, 2) changes in FOXM1 expression during anti-VEGF therapy and its potential as a biomarker of survival, and 3) TME change in accordance with the neuroradiographic response during Bev therapy.

## Methods

### Patient Characteristics and Treatment Protocol

The present study used 18 paired surgical samples from nine patients with newly diagnosed GBM obtained from surgery at the time of initial and recurrent tumors, including nine tumor samples obtained from surgery following neoBev, eight tumor samples obtained from autopsy, and one recurrent tumor. Four of these nine patients were included in the Japan Registry of Clinical Trials (jRCT1031180233).

All patients were treated with preoperative neoBev at a dose of 10 mg/kg on day 0. Surgical resection was performed 3–4 weeks after neoBev. Concomitant RT and TMZ were commenced more than 2 weeks after surgery. Maintenance treatment with TMZ began more than 4 weeks after completion of RT at a starting dose of 150 mg/m^2^ for 5 consecutive days of a 28-day cycle. Bev (at a dose of 10 mg/kg) concomitant with TMZ (every 4 weeks at a dose of 150 mg/m^2^) was readministered every 2 weeks at the time of recurrence and continued until reprogression or beyond reprogression in tolerant patients. The mean number of cycles of Bev was 16.1 (range, 7~38 cycles).

### Neuroradiological Assessment

The tumor volumes of T1-weighted images with gadolinium enhancement (T1Gd) or fluid-attenuated inversion recovery (FLAIR) were estimated by the sum of each slice on neuroimages and multiplication of longitudinal and transverse slices. Tumor volume was assessed by the sum of perpendicular diameters as previously described (23). The tumor regression rate with neoBev was evaluated by the change in tumor volume before and after treatment. Patterns of recurrence after Bev therapy were classified as cT1 flare-up, T2-diffuse, or T2-circumscribed as previously described (3, 24).

Briefly, the cT1 flare-up is characterized by an initial decrease in contrast enhancement (CE) on T1-weighted images after treatment initiation and an increase (flare-up) of CE again at tumor progression. T2 signal stays stable or increased. T2-diffuse is characterized by a signal increase on T2-weighted images with a poorly defined border despite the fact that CE on T1-weighted images remains decreased. Hypointensity on T1-weighted images is faint and disproportionally smaller than T2 hyperintensity. T2-circumscribed is characterized by a signal increase on T2-weighted images with a bulky structure and sharp borders that correspond to a T1 hypointense signal. CE on T1-weighted images remains decreased, or only a few faintly speckled CE lesions are visible.

### Immunohistochemical Analyses

Immunohistochemical analyses were performed on 4-µm sections of formalin-fixed, paraffin-embedded tissue from 18 tumors. Sections were stained with anti-FOXM1 antibody (1:250, #ab207298, abcam), anti-hypoxia-inducible factor-1 alpha (HIF-1α) antibody (1:100, #ab82832, Dako), anti-nestin antibody (1:1,000, MAB5326, Chemical), anti-carbonic anhydrase 9 (CA9) antibody (1:1,000, #ab15086, abcam), and anti-CD34 antibody (1:100, M7165, abcam). Antigen retrieval was performed in 10 mM citrate buffer (pH 6.0) using an autoclave for FOXM1, HIF-1α, and CD34 staining. Immunohistochemical staining was assessed by three authors (JT, NF, and TT) who were blinded to the clinical information, and the results of consensus among these authors were reported.

Immunohistochemical findings were assessed as previously described (5, 25, 26). For FOXM1 quantitative evaluation, the percentage of tumor nuclei reactive to FOXM1 antibody was estimated following examination of a middle-power field (×200) using the software Gunma labeling index (27). The expression of nestin was assessed as a positive cell ratio analyzed in five high-power fields (×400) and calculated as the mean value of [(positive cells/positive cell + negative cell) × 100] from five areas. The expression of HIF-1α was predominantly detected in the nuclei of tumor cells around sites of necrosis and was also found in tumor cells not directly adjacent to necrotic areas. The degree of expression was assessed as follows: ++, expression in >10% of tumor cells; +, expression in ≤10% of tumor cells; −, negative staining. The membranous expression of CA9 was predominantly found in perinecrotic tumor cells as previously reported (28). The degree of expression was assessed as follows: ++, universal strong expression around necrotic regions; +, occasional expression (typically around necrotic regions); −, negative staining. For quantitative evaluation of CD34+ vessels, the stained sections were screened in a low-power field (×40), and five middle-power fields (×200) with the most dense spots were assessed. The mean MVD in these areas was determined using Fiji software (version 2.0.0-re-69/1.52p) (29).

### Statistical Analyses

Continuous data are described as the mean ± standard deviation, median, and interquartile range, and categorical data as numbers and percentages. The Mann–Whitney U test and Wilcoxon signed rank test were used for comparison of continuous data between two groups. Fisher’s exact test was used to determine if non-random associations were present between two categorical variables. All p-values were two-sided with the significance level set to ≤0.05. Statistical analyses were performed with STATA 14 (Stata Corp. LP, College Station, TX, USA).

## Results

### Description of the Present Cohort

The characteristics of the patients in the present study are summarized in **Table 1**. Patients who were enrolled in the present study consisted of eight men and one woman with a mean age of 65.6 years (range, 50–78 years). Histological findings revealed that all tumors were diagnosed as GBM, isocitrate dehydrogenase (IDH)-wild type. Median tumor regression rates after neoBev were 38% and 54% on T1Gd and FLAIR magnetic resonance imaging (MRI), respectively. Median progression-free survival (PFS) (the interval from initial Bev administration to recurrence) was 9.8 months. Median overall survival (OS) (the interval from initial Bev to death) was 16.6 months.

**Table 1:** Patient characteristics.

		Total (n = 9)	
		No. of Patients	(%)
**Age (years)**	Mean	65.6	
	SD	10.8	
**Sex**	Women	1	11.1
	Men	8	88.9
**Neuroradiographic response after neoBev**			
T1Gd	Median	38%	
	IQR	15-56	
FLAIR	Median	54%	
	IQR	27-63	
**Recurrence pattern**	Nonresponder	0	0
	T2-circumscribed	2	22.2
	T2-diffuse	2	22.2
	cT1 flare-up	5	55.6
**PFS (months)**	Mean	9.8	
	SD	4.6	
**OS (months)**	Mean	16.6	
	SD	5.1	

FLAIR, fluid-attenuated inversion recovery; neoBev, neoadjuvant bevacizumab; OS, overall survival; PFS, progression-free survival; SD, standard deviation; T1Gd, T1-weighted image with gadolinium enhancement.

### Illustrative Neuroimages After NeoBev

**Figure 1:**
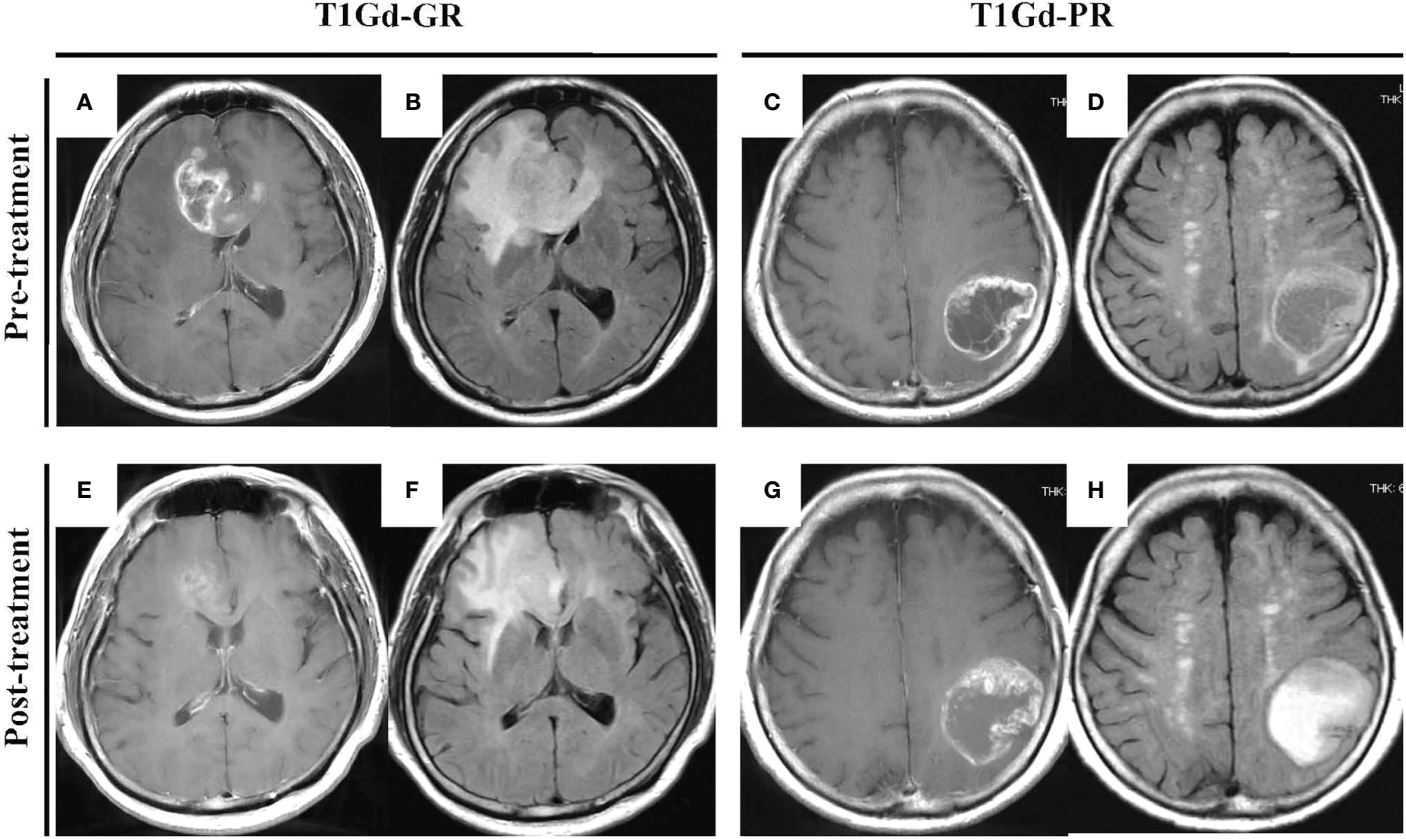
“Typical” GBM demonstrating a huge enhanced tumor with perifocal edema before **(A–D)** and after **(E–H)** neoBev. Regression rates of T1Gd-GR **(A, E)**, T1Gd-PR **(C, G)**, FLAIR-GR **(B, F)**, and FLAIR-PR **(D, H)** after neoBev were −61%, −14%, −71%, and −26%, respectively. FLAIR, fluid-attenuated inversion recovery; GBM, glioblastoma; GR, good rensponder; PR, poor responder; T1Gd, T1-weighted images with gadolinium enhancement.

Patients receiving neoBev were selected according to MRI findings that represented “typical” GBM including a ring-enhanced tumor (**Figures 1A, B**) with perifocal edema (**Figures 1C, D**). After a single dose of Bev (10 mg/kg), the tumor and the perifocal edema regressed as shown by a representative maximal (**Figures 1E, F**) and minimal response (**Figures 1G, H**) 2 weeks after treatment.

### Typical Histological Findings of “Pseudo-Papillary Structures” at the Time of Recurrence After NeoBev Demonstrating Colocalization of FOXM1, Nestin, CA9, and HIF-1α Expression

**Figure 2:**
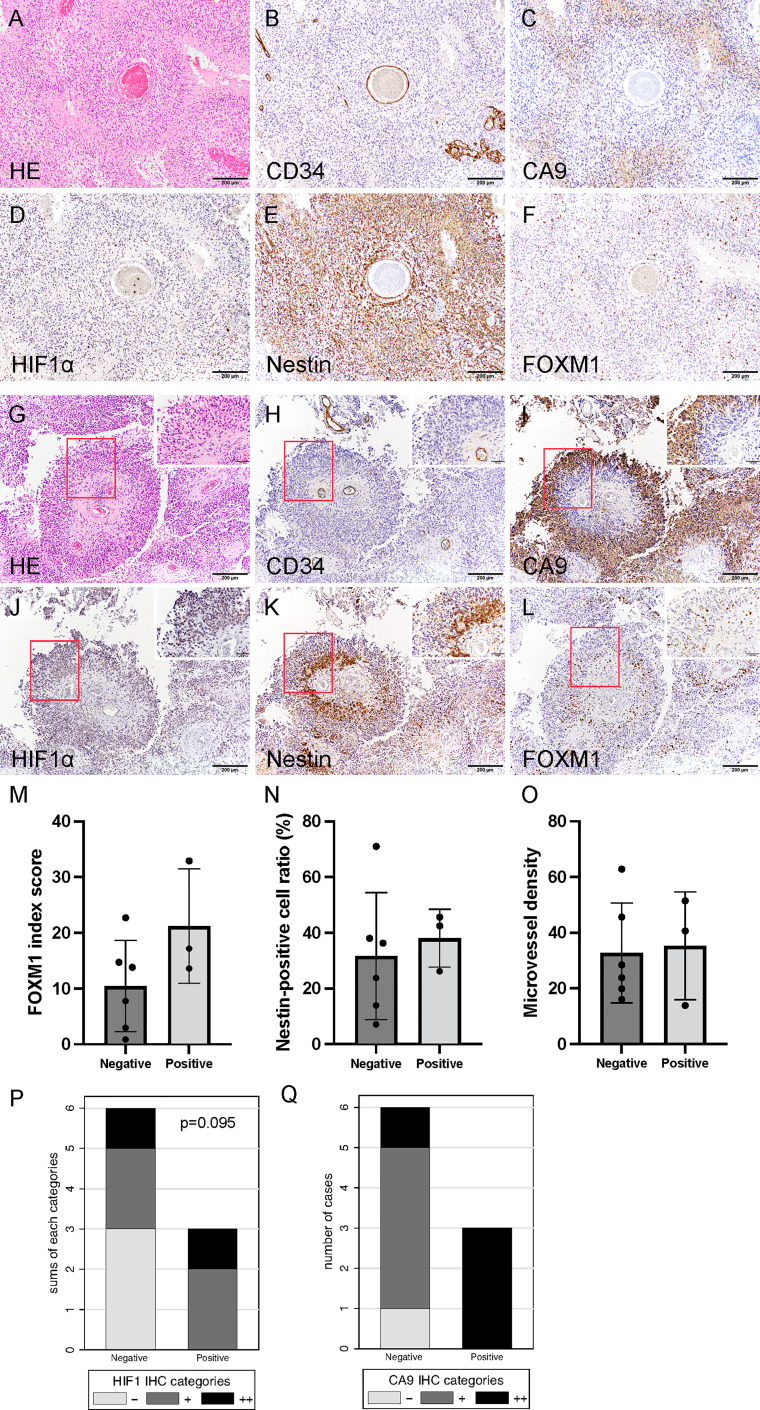
Histological finding of initial tumors revealing that tumor cells predominantly accumulated around the vessels (so-called vascular co-option). Hematoxylin and eosin staining **(A)** and CD34 **(B)**, CA9 **(C)**, HIF-1α **(D)**, nestin **(E)**, and FOXM1 expression **(F)**. Photomicrograph of immunohistochemistry (×200) (bar = 100 μm). Note that CA9 expression was found in the perinecrotic tumor cells, and the expression of HIF-1α, nestin, and FOXM1 was widely distributed. Typical histological findings of “pseudo-papillary” structures resembling “vascular co-option” in recurrent tumors. Hematoxylin and eosin staining **(G)** and CD34 **(H)**, CA9 **(I)**, HIF-1α **(J)**, nestin **(K)**, and FOXM1 expression **(L)**. Photomicrograph of immunohistochemistry (×200; bar = 100 μm); (×400; bar = 100 μm). Note that colocalization of FOXM1- and HIF-1α-positive cells was prominent in the perivascular area. Comparison of expression of FOXM1, MVD, and tumor oxygenation between the presence and absence of “pseudo-papillary” structures. FOXM1 **(M)**, nestin **(N)**, MVD **(O)**, CA9 **(P)**, and HIF-1α expression **(Q)**. Error bar; standard deviation. CA9, carbonic anhydrase 9; FOXM1, Forkhead box M1; HIF-1α, hypoxia inducible factor-1 alpha; MVD, microvessel density.

Histological findings of initial tumors after neoBev demonstrated that typical glomeruloid microvasculature was seldom observed. Tumor cells predominantly accumulated around the vessels (the so-called vascular co-option), and CD34+ cells were observed along the vessel walls (**Figures 2A, B**). The expression of CA9 was predominantly found in perinecrotic tumor cells (**Figure 2C**), but the positive expression of HIF-1α, nestin, and FOXM1 was widely distributed (**Figures 2F**).

Round-shaped tumor cells clustered adjacent to the blood vessels were further surrounded by spindle-shaped tumor cells (**Figures 2G, H**). We defined these characteristic histological findings as “pseudo-papillary” structures that were seen only in recurrent tumors but not in initial tumors. On the whole sections, these structures were observed in three out of nine recurrent tumors (33%).

Spindle-shaped cells were CA9+, and HIF-1α was strongly positive away from the blood vessels (**Figures 2I, J**). The distance from the blood vessel to the CA9+ cells was approximately 150 μm (data not shown). Interestingly, we noted a discrepancy in which the cells in the outer part of “pseudo-papillary” structures were CA9+/HIF-1α+, whereas the cells in the inner part of the structures were CA9−/HIF-1α+ (**Figures 2I, J**).

In the inner part of “pseudo-papillary” structures, nestin+ and FOXM1+ cells were clustered in round-shaped tumor cells adjacent to the blood vessels (**Figures 2K, L**), suggesting that proliferating GSCs adjacent to the microvasculature were surrounded by hypoxic tumor cells in recurrent tumors at the time of Bev failure.

MVD was not different between tumors with and without “pseudo-papillary” structures. However, the expression of FOXM1, nestin, and hypoxic markers including CA9 and HIF-1α tended to be higher in recurrent tumors with “pseudo-papillary” structures (**Figures 2M–Q**).

Patient characteristics, neuroradiological response rate after neoBev, recurrent pattern after Bev failure, and extent of resection were compared between the presence and absence of “pseudo-papillary” structures. The clinical outcome and clinical parameters were not significantly different (**Table 2**).

**Table 2:** Comparison of clinical characteristics between positive and negative “pseudo-papillary” structures.

				Pseudo-papillary structures		
		Positive (n = 3)		Negative (n = 6)		p value
		No. of Patients	(%)	No. of Patients	(%)	
**Age (years)**	Mean	57.7		69.5		0.121*
	SD	10.8		9.2		
**Sex**	Women	0.0	0.0	1	16.7	1.000†
	Men	3.0	100.0	5	83.3	
**Neuroradiographic response after neoBev**						
T1Gd	Mean	12.7%		41.7%		0.302*
	SD	37.8		23.1		
FLAIR	Mean	47.3%		46.7%		0.796*
	SD	20.8		20.2		
**Surgical removal**	Total	2	66.7	4	66.7	1.000†
	Not total	1	33.3	2	33.3	
**Recurrence pattern**	Not described	0	0	0	0.0	0.762†
	T2-circumscribed	0	0	2	33.3	
	T2-diffuse	0	0	2	33.3	
	cT1 flare-up	3	100	2	33.3	
**PFS (months)**	Mean	8.7		10.4		0.663‡
	SD	1.5		5.6		
**OS (months)**	Mean	15.7		17.1		0.927‡
	SD	5.1		5.8		
**FOXM1 index score at the time of recurrence**						
	Mean	21.2		10.3		0.197*
	SD	10.3		8.4		

*Mann–Whitney U test.

†Fisher’s exact test.

‡Log-rank test.

### Comparison of Tumor Vascularity and Tumor Oxygenation Between Initial Tumors After NeoBev and Recurrent Tumors After NeoBev

**Figure 3:**
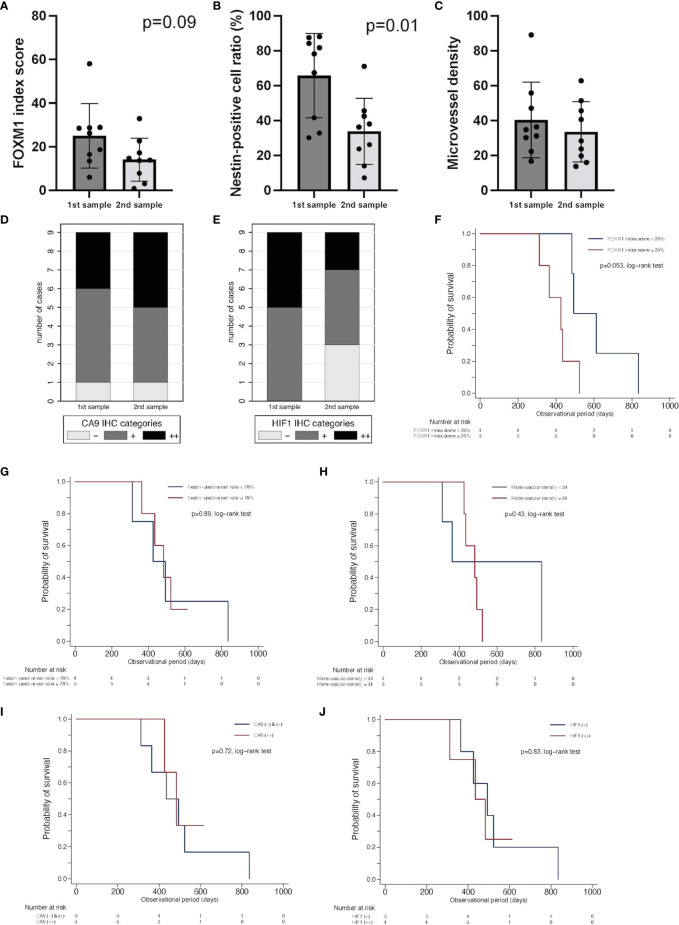
Comparison of expression of FOXM1, tumor vascularity, and tumor oxygenation between initial tumors after neoBev and recurrent tumors after neoBev. FOXM1 **(A)**, nestin **(B)**, MVD **(C)**, CA9 **(D)**, and HIF-1α expression **(E)**. Error bar; standard deviation. Kaplan–Meier survival curves showing overall survival stratified by labeling index of FOXM1 expression (p = 0.053, log-rank test) **(F)**, labeling index of nestin expression (p = 0.89, log-rank test) **(G)**, microvascular density (p = 0.43, log-rank test) **(H)**, qualitative reaction of CA9 expression (p = 0.72, log-rank test) **(I)**, and qualitative reaction of HIF-1α expression (p = 0.83, log-rank test) **(J)**. CA9, carbonic anhydrase 9; FOXM1, Forkhead box M1; HIF-1α, hypoxia inducible factor-1 alpha; MVD, microvessel density; neoBev, neoadjuvant bevacizumab.

To assess vascular density and stemness in accordance with tumor oxygenation, the expression levels of FOXM1, nestin, CD34, CA9, and HIF-1α were analyzed with immunohistochemistry using paired samples from the same patients treated with neoBev followed by surgery, RT, TMZ, and TMZ/Bev combined therapy, and then salvage surgery or autopsy at the time of recurrence after Bev (**Figure 3**).

FOXM1 tended to decrease at the time of recurrence (**Figure 3A**), and nestin was found to be significantly decreased at the time of recurrence (**Figure 3B**). MVD diminished during Bev therapy, but the difference was not statistically significant (**Figure 3C**). The expression of CA9 (++) was slightly higher in recurrent tumors compared with initial tumors, but HIF-1α expression decreased in recurrent tumors (**Figures 3D, E**). To determine whether or not these parameters have prognostic significance, we investigated CD34, CA9, HIF-1a, nestin, and FOXM1. We divided the current cohort into two groups according to the median index score of those parameters in initial specimens. FOXM1 low-expression tumors tended to occur in patients with a better prognosis than FOXM1 high-expression tumors (p = 0.053, log-rank test) (**Figure 3F**). Whereas other parameters including expression levels of nestin (p = 0.89, log-rank test) (**Figure 3G**), microvascular density as quantified by CD34 positivity (p = 0.43, log-rank test) (**Figure 3H**), qualitative reaction of CA9 (p = 0.72, log-rank test) (**Figure 3I**), and qualitative reaction of HIF-1α (p = 0.83, log-rank test) (**Figure 3J**) were not associated with OS in the current cohort.

### T1Gd-GR vs. T1Gd-PR in the Tumor Microenvironment Including Tumor Oxygenation, Stemness, and Tumor Vascularity

To analyze the correlation between the TME assessed with immunohistochemistry and responsiveness to neoBev assessed with T1Gd and FLAIR, the expression of FOXM1, nestin, CD34, CA9, and HIF-1α was compared between GR and PR after neoBev.

We divided this cohort into two groups according to the imaging neoadjuvant therapy response rate. Thus, T1Gd good responders (T1Gd-GRs) and T1Gd poor responders (T1Gd-PRs) were defined as having a response rate of ≥35% and <35%, respectively.

**Figure 4:**
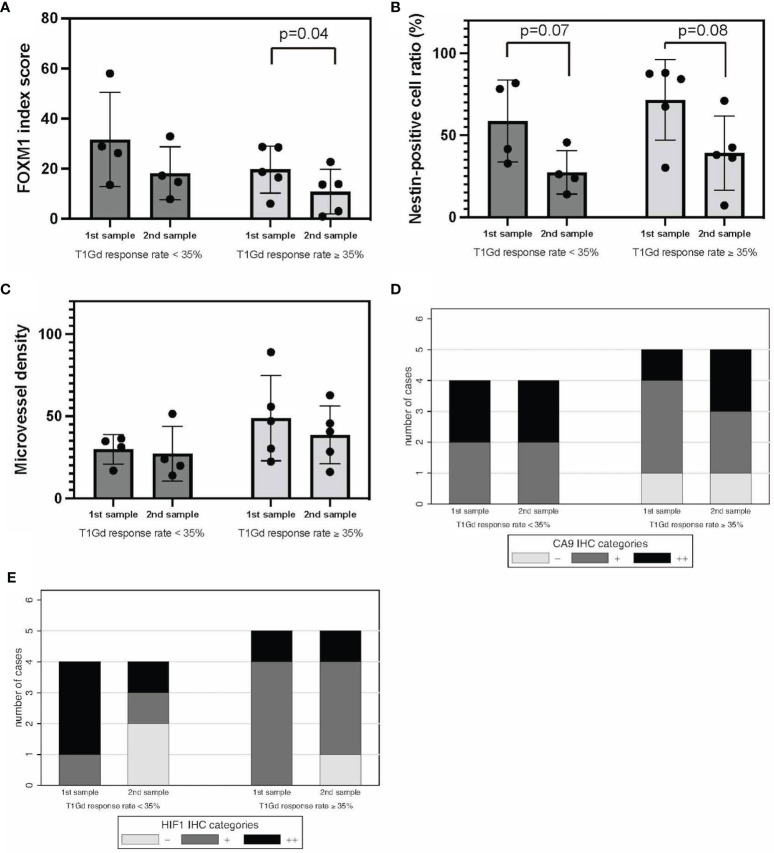
T1Gd-GRs vs. T1Gd-PRs in tumor oxygenation, stemness, and immunological TME. FOXM1 **(A)**, nestin **(B)**, MVD **(C)**, CA9 **(D)**, and HIF-1α expression **(E)**. Error bar; standard deviation. CA9, carbonic anhydrase 9; FOXM1, Forkhead box M1; GR, good rensponder; HIF-1α, hypoxia inducible factor-1 alpha; MVD, microvessel density; PR, poor responder; TME, tumor microenvironment; T1Gd, T1-weighted images with gadolinium enhancement.

Regarding stemness, FOXM1 in recurrent tumors was significantly decreased in T1Gd-GRs, whereas no significant difference was found in T1Gd-PRs (**Figure 4A**). Nestin expression in recurrent tumors tended to be decreased in both T1Gd-GRs and PRs (**Figure 4B**). MVD showed no significant difference between T1Gd-GRs and T1Gd-PRs (**Figure 4C**).

Regarding the hypoxic TME, comparing initial tumors, the expression of CA9 and HIF-1α was higher in T1Gd-PRs, and tumor oxygenation was frequently observed in T1Gd-GRs, although no significant difference was found between the two groups (**Figures 4D, E**). Thus, these results suggested that the responsiveness to neoBev determined on T1Gd may reflect tumor oxygenation.

### FLAIR-GR vs. FLAIR-PR in the Tumor Microenvironment Including Tumor Oxygenation, Stemness, and Tumor Vascularity

**Figure 5:**
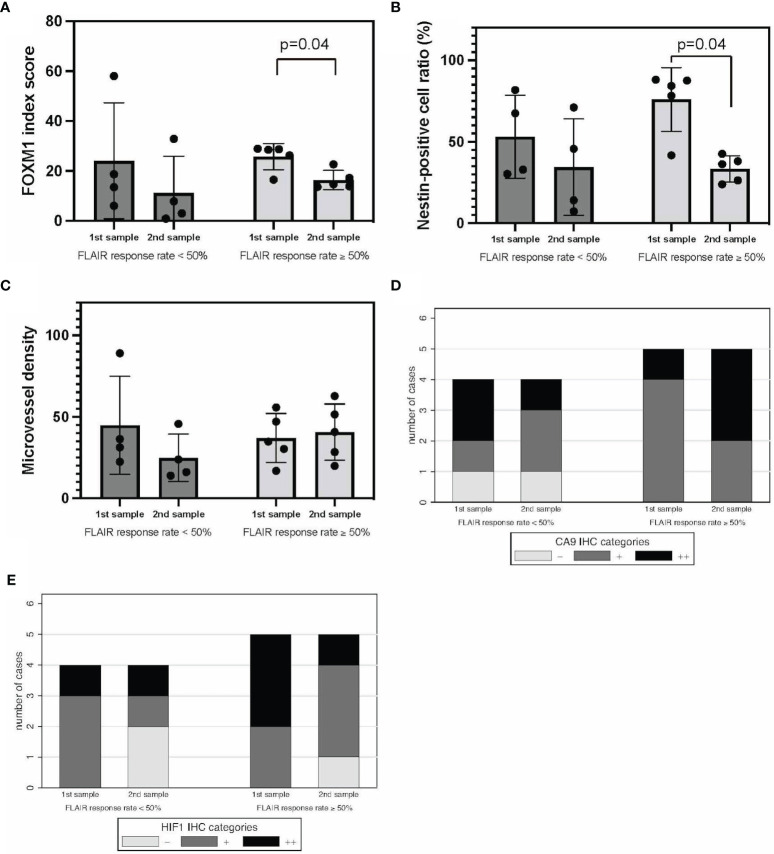
FLAIR-GRs vs. FLAIR-PRs in tumor oxygenation, stemness, and immunological TME. FOXM1 **(A)**, nestin **(B)**, MVD **(C)**, CA9 **(D)**, and HIF-1α expression **(E)**. Error bar; standard deviation. CA9, carbonic anhydrase 9; FLAIR, fluid-attenuated inversion recovery; FOXM1, Forkhead box M1; GR, good rensponder; HIF-1α, hypoxia inducible factor-1 alpha; MVD, microvessel density; PR, poor responder; TME, tumor microenvironment.

FLAIR good responders (FLAIR-GRs) and FLAIR poor responders (FLAIR-PRs) were defined as having a response rate of ≥50% and <50%, respectively. No significant difference in FOXM1 or nestin expression was found between FLAIR-GRs and FLAIR-PRs in the initial tumors. However, the expression of both FOXM1 and nestin significantly decreased in FLAIR-GRs at the time of recurrence (**Figures 5A, B**). MVD showed no significant difference between FLAIR-GR and FLAIR-PR (**Figure 5C**).

Regarding the hypoxic TME, CA9 and HIF-1α expression tended to decrease in FLAIR-PRs compared with that in FLAIR-GRs in both initial and recurrent tumors (**Figures 5D, E**). No significant difference was found between groups. Thus, neuroradiographic response on T1Gd and FLAIR to neoBev may illustrate opposite changes in both CA9 and HIF-1α expressions.

### Recurrence Pattern in Tumor Oxygenation, Stemness, and Immunological Tumor Microenvironment

**Figure 6:**
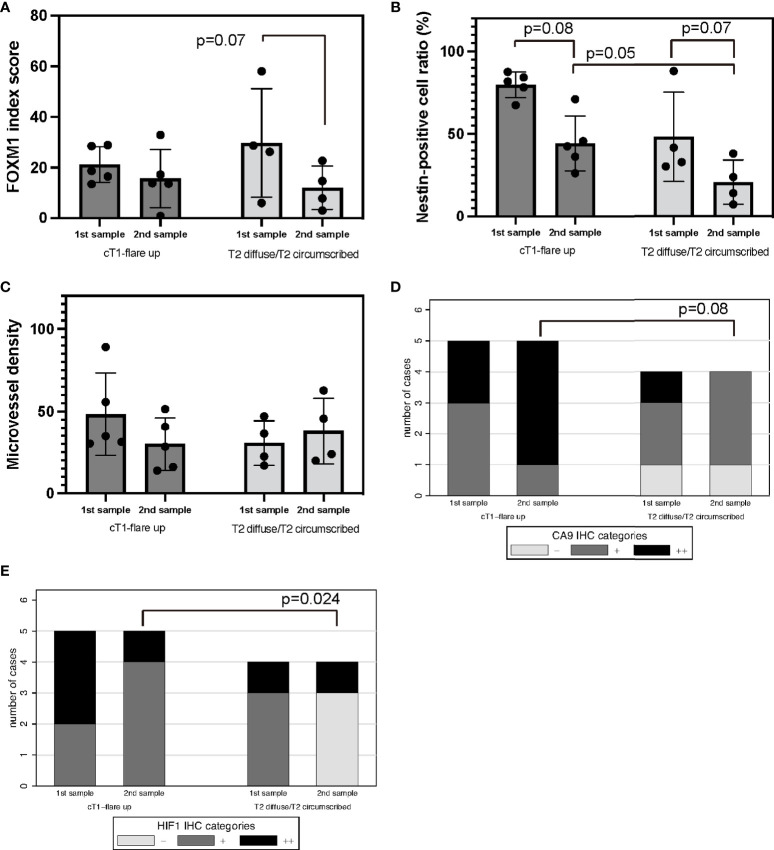
Recurrence pattern on MRI after Bev therapy; “cT1 flare-up” vs. “T2-diffuse/T2-circumscribed” in tumor oxygenation, stemness, and immunological TME. Recurrent GBM after Bev therapy in cT1 flare-up and T2-diffuse GBM. FOXM1 **(A)**, nestin **(B)**, MVD **(C)**, CA9 **(D)**, and HIF-1α expression **(E)**. Error bar; standard deviation. Bev, bevacizumab; CA9, carbonic anhydrase 9; FOXM1, Forkhead box M1; GBM, glioblastoma; HIF-1α, hypoxia inducible factor-1 alpha; MRI, magnetic resonance imaging; MVD, microvessel density; TME, tumor microenvironment.

To analyze the correlation between the TME assessed with immunohistochemistry and the recurrence pattern after Bev therapy as previously described (24), the expression of FOXM1, nestin, CD34, CA9, and HIF-1α was compared between “T1 flare-up” and “T2-circumscribed/T2-diffuse” patterns. FOXM1 expression tended to decrease in the “T2-circumscribed/T2-diffuse” pattern at the time of recurrence (**Figure 6A**). Nestin expression was reduced at recurrence in both patterns, but this reduction was not statistically significant (**Figure 6B**).

MVD was not significantly different between the two groups (**Figure 6C**).

Regarding the hypoxic TME, CA9 and HIF-1α expression decreased in the “T2-circumscribed/T2-diffuse” pattern compared with that in the “T1 flare-up” pattern in recurrent tumors (**Figures 6D, E**). HIF-1α expression in particular significantly indicated oxygenation (p = 0.024, Fisher’s exact test). Thus, the recurrence pattern after Bev therapy may be correlated with tumor oxygenation after multidisciplinary treatment of GBM including Bev.

## Discussion

### Histological Assessment and Bevacizumab Responsiveness

Our previous reports described histological findings of GBM with resistance to Bev (refractory-Bev) because Bev is usually administered to patients with recurrent GBM (3, 26). Over the past decade, the measurement of tumor vascularity with MVD has been suggested to provide histological assessment, be correlated with the invasiveness of cancer, and provide prognostic information (30). In addition, histological assessment of both tumor oxygenation and angiogenesis may be useful for the assessment of the effectiveness of antiangiogenic therapy such as Bev. However, in the present study, no relationship between hypoxia and MVD was found, as previously described (31). More reliable histological parameters are required.

Vascular co-option has received particular attention as a major mechanism of resistance to antiangiogenic treatment (32–34). Vascular co-option is a mechanism by which tumors incorporate the existing vessels of the host organs, preserving the vascular scaffold of the surrounding tissue. Vascular co-option may be an adaptive mechanism that enables tumors to survive and progress when angiogenesis is inhibited. In the present study, “pseudo-papillary” structures containing nestin+/FOXM1+ cells in the perivascular niche and CA9/HIF-1α positivity in the area surrounding stem cell accumulation resembled co-opted tumor vessels and were observed in refractory-Bev (**Figure 2**). Recurrent GBM may exploit vascular co-option as a strategy to escape anti-VEGF treatment. Optimization of anticancer therapy should consider the importance of hypoxia as a master driver of tumor angiogenesis and immunoregulatory response.

In the present study, we found that “pseudo-papillary” structures were only present in recurrent tumors. Because they are not found in initial tumors after neoBev, this phenomenon may be the result of long-term anti-VEGF therapy. A peculiar point regarding “pseudo-papillary” structures is the discrepancy in the expression pattern between CA9 and HIF-1α and nestin+/FOXM1+ cells revealed by immunohistochemical staining. The outer cells were CA9+/HIF-1α+/nestin−/FOXM1−, and the inner cells were CA9−/HIF-1α+/nestin+/FOXM1+. The discrepancy between CA9 and HIF-1α expression has been reported by Kaluz et al. (35) and reproduced *in vivo*. HIF-1α expression is induced not only in hypoxic conditions but also for various reasons, especially in RAS and phosphoinositol-3 kinase (PI3K) hyperactivation (35). Furthermore, nestin+ GSCs located in the perivascular niche adjacent to the blood vessels may survive *via* PI3K pathway hyperactivation after RT (36).

In addition, the distance of more than 150 μm of cells from the blood vessels indicates that the cells are in a hypoxic TME (37–39). The present study demonstrated that CA9+ cells were present at 150 μm from the blood vessels in the “pseudo-papillary” structures, which is in agreement with a previous theory. CA9 is also induced by downregulation of tumor suppressor genes such as *p53* and *PTEN*, induction of oncogenic pathways including PI3K, and other environmental conditions including acidosis and glucose deprivation (35). These findings suggest that the inner cells of “pseudo-papillary” structures may consist of nestin+ GSCs with enhanced PI3K activity in a hypoxic environment as evidenced by high expression of HIF-1α. In other words, “pseudo-papillary” structures may reflect a mechanism of resistance of tumor cells to anti-VEGF therapy by producing a favorable TME for GSCs.

### FOXM1 as a Potential Biomarker for Survival

In the present study, we investigated FOXM1 and nestin expression and found that changes in the expression of both were similar during Bev therapy. FOXM1 is involved in tumorigenesis and transformation of normal astrocytes, reflects the histological malignancy of glioma, and is proposed to be a surrogate marker for OS (20, 22). Interestingly, FOXM1 also binds the *VEGF* promoter and contributes to the angiogenesis and growth of GSCs in GBM by upregulation of VEGF (21). Thus, FOXM1 may be a marker for GSCs with growth potential and a prediction biomarker for survival and may thus be useful for optimizing VEGF-targeted antiangiogenic therapy including neoBev in newly diagnosed GBM.

To verify this hypothesis, the present cohort was restricted to preoperative and recurrent GBM after neoBev in the same patients. The cohort was divided into high and low levels of FOXM1 in initial samples to compare OS. Patients in the group with lower expression of FOXM1 after neoBev tended to show better OS than those with higher expression of FOXM1, suggesting that FOXM1 as a marker of proliferating GSCs may be a predictive factor for long-term survival during Bev therapy for newly diagnosed GBM. No previous studies have investigated this point.

An additional interesting finding about FOXM1 in the present study was that FOXM1 expression tended to decrease in recurrent samples and was significantly decreased in T1Gd-GRs and FLAIR-GRs. This finding is contrary to that of Zhang et al. (22) who found that FOXM1 expression is upregulated in recurrent GBM samples. This inconsistency may be due to the presence or absence of anti-VEGF therapy. The previous study by Zhang et al. (22) included 38 pairs of primary and recurrent GBM tumor samples, and all 38 patients received concomitant RT and TMZ after surgery. The present study included nine patients who received neoBev, followed by TMZ plus RT after surgery, and then subsequent Bev. Hence, anti-VEGF therapy for GBM may inhibit FOXM1 expression for a long period of time up to the point of recurrence, or recurrent GBM may be able to proliferate without FOXM1 upregulation during anti-VEGF therapy. Further investigation is needed to address this question.

### Neuroimaging and Bevacizumab Responsiveness

With regard to the therapeutic response to Bev assessed with neuroimaging, the type of radiological progression after Bev therapy and its relationship to PFS and OS were investigated. Newly diagnosed GBM responded to Bev therapy, but the therapeutic effects are usually transient. GBM progression during Bev therapy can exhibit non-enhancing T2-weighted image/FLAIR-bright growth with invasion or restricted enhancement with contrast medium. The difference between non-enhancing and enhancing lesions after Bev therapy in terms of PFS and OS is controversial (40, 41). Whether favorable and poor responsiveness during Bev therapy determines the clinical outcome is still controversial. Previous studies in the cohort of newly diagnosed and recurrent GBM concluded that complete resolution of CE during treatment is a favorable factor for the clinical outcome (40, 42).

In contrast, Ellingson et al. (43) insisted that objective response rates are not clinically meaningful in newly diagnosed GBM and suggested that a measure of early PFS or treatment failure rates during the maintenance phase may be extremely useful for predicting the long-term outcome. Despite this observation, a survival difference in patients with growing vs. shrinking tumors was not maintained, suggesting that this may not be the most sensitive method for evaluating efficacy and predicting OS in newly diagnosed GBM (43). The mechanism of sustaining tumor dormancy is probably related to the TME and is an important issue to be investigated. Understanding the mechanism of sustaining tumor dormancy by comparing histological or molecular features between the effective and refractory phase during Bev therapy may be useful. Volumetric analyses as described above investigated newly diagnosed and recurrent GBM in different therapeutic situations of Bev combined with surgical resection followed by RT and TMZ. To the best of our knowledge, the present study is the first report to include an exploratory volumetric analysis during Bev treatment alone in newly diagnosed GBM.

One of the most important issues for comprehending the mechanism of Bev effectiveness and resistance is the variable TME from hypoxic and normoxic conditions with reversible alterations. Tumor oxygenation in relation to Bev effectiveness was demonstrated by neuroimaging using a representative hypoxia positron emission tomography (PET) tracer, ^18^F-fluoromisonidazole (FMISO) PET. According to a previous investigation regarding the association between FMISO PET findings and Bev treatment for high-grade glioma, recurrent gliomas with decreasing FMISO accumulation after short-term Bev application derive a survival benefit from Bev therapy (44). In addition, a correlation was found between FMISO uptake and HIF-1α/VEGF expression detected with immunohistochemistry in newly diagnosed GBM (45). These results suggested that tumor oxygenation was maintained during Bev effectiveness as evidenced by histological findings with support of neuroimaging.

In the present study, the difference in stemness and oxygenation during effectiveness and refractoriness was demonstrated with immunohistochemistry. In addition, responsiveness to neoBev was also represented by neuroradiological findings including T1Gd. A comparison of stemness/oxygenation of the TME assessed with FMISO PET and immunohistochemistry between the Bev-effective and Bev-refractory periods is of great interest.

A hypoxic TME causes resistance to Bev due to stem cell accumulation (46). We previously reported that Bev-effective GBM exhibits reduced hypoxia along with reduced infiltration of GSCs compared with naive-Bev GBM (3). Very few reports have demonstrated that tumor oxygenation is maintained during Bev effectiveness in GBM by histological analysis with molecular profiling.

We investigated the expression levels of hypoxic markers (CA9 and HIF-1α) and a GSC marker (nestin) using GBM samples obtained from three different settings including tumors before Bev therapy (naive-Bev), tumors resected following neoBev (effective-Bev), and recurrent tumors following Bev therapy (refractory-Bev) (3). Recurrent tumors after neoBev were not included in those studies. The clinical outcome following neoBev and the impact of a change in response on OS assessed with neuroradiological findings and the TME, including oxygenation and stemness with immunohistochemical analysis, has not been previously investigated. Thus, to confirm whether a change in the TME determines disease control during Bev therapy, paired samples between effective-Bev and refractory-Bev were compared using neuroradiological and histological analyses.

### Recurrent Pattern and Change in the Tumor Microenvironment After Bevacizumab Failure

Regarding molecular features when the tumor recurred during Bev therapy, DeLay et al. (41) showed that non-enhancing Bev-resistant GBM and enhancing Bev-resistant GBM have different molecular features and that the TME including vessel density and hypoxia is also different. Compared with paired samples before Bev therapy, non-enhancing Bev-resistant GBM exhibited reduced vessel density and increased hypoxia as evidenced by increased CA9 and HIF-1α staining. In contrast, enhancing Bev-resistant and naive-Bev GBM exhibited unchanged vessel density and hypoxia. Interestingly, VEGF/VEGF receptor expression was not altered in pre-Bev compared to post-Bev therapy tumors in their series. However, invasion molecules including integrin β1 were elevated in non-enhancing Bev-resistant GBM, indicating that neuroimaging reflects molecular profiling (41). In the present study, CA9 and HIF-1α expression decreased in the “T2-circumscribed/T2-diffuse” pattern compared with the “T1 flare-up” pattern in recurrent tumors (**Figure 6**). This result seemed to be inconsistent with the previous study by DeLay et al. (41), but the background was different in two ways. First, the present study focused on paired comparisons of GBM during effectiveness (effective-Bev) and refractoriness of Bev (refractory-Bev), whereas DeLay et al. (41) described naive-Bev and refractory-Bev. Second, the current study followed the definition of the recurrence pattern as described by Nowosielski et al. (24), whereas DeLay et al. (41) used the percentage of FLAIR-bright volume exhibiting T1Gd enhancement. Therefore, our results suggested that the growth potential was preserved in recurrent GBM with the “T2-circumscribed/T2-diffuse” pattern in the absence of severe hypoxia at the time of recurrence.

### Mechanism of Duration of Bevacizumab Effectiveness in Light of Metabolic Adaptation

According to a previous report (47), mutant epidermal growth factor receptor variant III (EGFR vIII) mutation and EGFR overexpression glioma cells impaired physiological adaptation to starvation and rendered cells sensitive to hypoxia-induced cell death. Theoretically, the activation of EGFR enhances vulnerability to hypoxia-inducing therapies *via* a decrease in Nicotinamide adenine dinucleotide phosphate (NAPDH) levels. Therefore, we should consider the possibility of the biological behavior of tumor cells related to their metabolism adaptation during Bev therapy.

As we indicated in the current and previous papers, Bev could change from normoxic during its effectiveness to hypoxic TME after its failure (3). The duration of Bev effectiveness might be associated with the status of EGFR overexpression or EGFR vIII mutation in GBM probably due to the impact of Bev on metabolism adaptation (48). To address these issues, further investigation is needed.

### Limitations

The present study using infrequently available clinical specimens has some limitations. The first limitation is the paucity of the number of paired samples from the same patients due to the extreme rarity of salvage surgery or autopsy for recurrent GBM after the failure of RT and TMZ with Bev. The second limitation is that the influence of RT and TMZ combined with Bev was not considered. The third limitation is that the present study evaluated surgically resectable large enhanced tumors with expanding perifocal edema as seen with neuroimaging, which adds bias.

The fourth limitation is that an initial favorable neuroradiographic response to neoBev on a FLAIR image does not always reflect prolonged PFS and OS, thus initial neuroradiological responsiveness after Bev therapy could not reflect a clinical benefit of Bev, especially for newly diagnosed GBM. Based on the present study, FOXM1 seemed to be a predictive prognostic biomarker, but it was not verified whether the expression level of this biomarker should determine the duration of Bev effectiveness and advantage of neoBev. Further investigation would be needed to solve this clinical question.

In summary, whether a visible favorable response on neuroimaging following Bev therapy predicts the clinical outcome is uncertain. However, this is the first report regarding an investigation of neuroradiographic response to neoBev associated with hypoxic and stem cell markers as evidenced by immunohistochemistry. Based on results in the present study demonstrating that Bev produced an oxygenated TME in addition to stemness inhibition, combination therapy with Bev and immunotherapy may contribute to improvements in the currently dismal clinical outcome of GBM.

## Conclusion

“Pseudo-papillary” structures were seen in recurrent GBM after anti-VEGF therapy. An interesting discrepancy in CA9 and HIF-1α expression in these “pseudo-papillary” structures was observed. A neuroradiographic response after neoBev may reflect the status of the TME including stemness and oxygenation. Bev may produce tumor oxygenation, leading to suppression of proliferation of GSCs. Results in the present study suggested that FOXM1 plays a potential role as a biomarker of survival during anti-VEGF therapy.

## Data Availability Statement

The raw data supporting the conclusions of this article will be made available by the authors without undue reservation.

## Ethics Statement

The studies involving human participants were reviewed and approved by the Ethical Committee of Jikei University School of Medicine. The patients/participants provided their written informed consent to participate in this study. Written informed consent was obtained from the individual(s) for the publication of any potentially identifiable images or data included in this article.

## Author Contributions

TT: design of the present study. JT, NF, and YY: data collection and analysis. RT, HS, and YA: article survey. KY, MM, MS, and YM: suggestions for the article. All authors contributed to the article and approved the submitted version.

## Funding

The study was supported by the Ministry of Education, Culture, Sports, Science and Technology and the Japan Society for Promotion of Science (KAKENHI) (Grant Number 21K09161).

## Conflict of Interest

The authors declare that the research was conducted in the absence of any commercial or financial relationships that could be construed as a potential conflict of interest.

## Publisher’s Note

All claims expressed in this article are solely those of the authors and do not necessarily represent those of their affiliated organizations, or those of the publisher, the editors and the reviewers. Any product that may be evaluated in this article, or claim that may be made by its manufacturer, is not guaranteed or endorsed by the publisher.
